# Interactive Effects of Climate and Large Herbivore Assemblage Drive Plant Functional Traits and Diversity

**DOI:** 10.3390/plants14081249

**Published:** 2025-04-20

**Authors:** Maggie Klope, Ruby Harris-Gavin, Stephanie Copeland, Devyn Orr, Hillary S. Young

**Affiliations:** Department of Ecology, Evolution, and Marine Biology, University of California Santa Barbara, Santa Barbara, CA 93106, USA; mmklope@ucsb.edu (M.K.); jrharrisgavin@ucsb.edu (R.H.-G.); scopeland@ucsb.edu (S.C.); devynorr@gmail.com (D.O.)

**Keywords:** plants, herbivores, functional traits, climate, community

## Abstract

Large herbivore communities are changing globally, with populations of wild herbivores generally declining while domestic herbivore populations are increasing, influencing ecosystem function along with the impacts of climate change. Manipulative experiments have rarely captured the interaction between patterns of large herbivore assemblage change and climatic conditions. This interaction may affect the functional traits and functional diversity of herbaceous communities; this requires investigation, as these metrics have been useful proxies for ecosystem function. We used a large herbivore exclosure experiment replicated along a topo-climatic gradient to explore the interaction between climate and herbivore assemblage on community-level functional traits and the functional diversity of herbaceous plant understories. Our findings demonstrate interacting effects between large herbivore assemblages and climate. We found a shift from drought-tolerant traits to drought-avoidant traits with increasing aridity, specifically with regard to plant leaf area and specific leaf area. We also determined that plant community responses to grazing changed from an herbivore avoidance strategy at drier sites to a more herbivore-tolerant strategy at wetter sites. We observed that the effects of herbivores on community-level traits can sometimes counteract those of climate. Finally, we found that cattle and large wild herbivores can differ in the magnitude and direction of effects on functional traits and diversity.

## 1. Introduction

Large herbivores profoundly shape herbaceous plant communities, impacting plant abundance, biomass, survival, and reproduction [1,2,3]. These effects have been extensively studied using large herbivore exclosures to simulate the loss or displacement of native large herbivore communities [4]. However, while at the global scale, large wild herbivore communities are generally declining [5], they are often replaced by domestic livestock at densities far exceeding the historic densities of their wild counterparts [6]. While these changes in herbivore density and identity can have a substantial effect on plant communities and ecosystem function [7,8,9], few experimental studies explicitly focus on realistic changes in herbivore assemblages by manipulating domestic and wild herbivores simultaneously [7]. Similarly, although it is well established that the effects of herbivores change across climatic contexts, there are few, if any, experiments that have investigated how the effects of this realistic change in herbivore assemblages may vary across climate contexts. This is likely due to the logistical challenges of creating an ecological experiment which manipulates both herbivore assemblages and climate.

In this study, we explore the interactive effects of climate and changes in large herbivore (hereafter referred to as “herbivore”) assemblages on plant functional traits and functional diversity. Understanding functional traits of plant responses to disturbance such as large herbivore loss and climate change is crucial because these traits—such as seed size, resprouting ability, growth rate, floral size, and water investment strategies—help predict how ecosystems recover and which species dominate after such disturbances [10,11]. This trait-based approach allows for more generalizable and mechanistic insights into community assembly and ecosystem resilience across different environments [12]. Functional trait diversity is also important to explore, as it captures the range of ecological strategies within a community, helping predict how ecosystems respond to environmental changes [13]. Critically, together these metrics also offer a powerful tool to understand the impacts of global change because plant functional traits and diversity are often tightly tied to ecosystem functions [12,14,15,16,17,18,19,20]. In this context, plant functional traits and functional diversity can provide insights into how both changes in climatic contexts and herbivore abundance or identity may alter plant communities and the services they provide [12,21,22], potentially helping resolve some of the highly variable effects that changing large herbivores have on ecosystem function across contexts [7].

The impacts of herbivores on plant functional traits have been studied in a variety of systems, and plant responses are typically placed into one of two strategies: herbivore avoidance and herbivore tolerance. In areas with a long history of grazing or with high grazing intensity, plant communities typically have a greater proportion of herbivore-tolerant species [23,24]. These species compensate for the frequent removal of biomass by quickly growing leaves and are characterized by high specific leaf area (SLA), low leaf dry matter content (LDMC), and high leaf nitrogen concentration (LNC) (Table 1) [22,25]. With low levels of herbivory, there are often more plant species with herbivore avoidance traits [26], which are characterized by their shorter stature, smaller leaves, lower SLA, and higher LDMC [27]. However, the tradeoff between herbivore avoidance and tolerance strategies is dependent on herbivore identity and selectivity, ecosystem productivity, plant community composition, and nutrient availability [28,29,30,31].

Existing research on the impacts of grazing on functional diversity has generated mixed results; functional diversity has been shown to be positively correlated [32,33,34,35], negatively correlated [36,37,38], and uncorrelated with herbivore presence [35,38,39]. Such differing responses may be due to the variations in grazing intensity and timing across studies, both of which can moderate the strength of plant community responses to grazing [40,41,42], functional traits, and diversity change [31,43]. Additionally, herbivore identity can affect the magnitude and direction of responses of plant community species composition and diversity as well as functional traits and functional diversity [30]. Therefore, to accurately predict the effects of changing herbivore assemblages on plant functional traits and diversity in an anthropogenic landscape, it is important to study the impacts of both native herbivore and livestock species. However, while studies have examined the differential effects of specific livestock species on plant functional traits and diversity, few studies have explicitly compared differences in these metrics between native and introduced herbivores in the same system (but see [9,44,45]).

Plant functional traits and diversity are also strongly influenced by climatic conditions. At a global level, plant functional traits vary with climate [46,47,48], while at a local level, differing responses to climate are seen based on plant communities and functional groups [30,49,50,51]. Globally, in areas with moderately high levels of aridity, vascular plants generally experience decreased leaf area (LA), SLA, and LNC (Table 1) [48,52,53]. However, in extremely arid conditions and Mediterranean climates, the opposite pattern may occur [54]. This is due to an increased amount of drought-tolerant species in these systems, which are characterized by high LA and SLA, whereas more mesic systems typically have more drought-avoidant species with low values of LA and SLA. Climate effects on the functional diversity of herbaceous communities are variable. Some studies have shown functional diversity to be higher at arid sites [36,55] and to increase with long-term drought [56], while others have shown that it may be uncoupled from climate [38,57]; however, the latter studies may not have sampled sufficiently arid climates to detect alterations to functional diversity.

Plant community responses to herbivores are also known to be dependent on climate [4,9,36,41,45]. Notably, evidence and theory suggest that aridity and grazing often drive similar shifts in plant functional traits, favoring stress-tolerant, slow-growing species with traits like small stature, tough leaves, and high root-to-shoot ratios [1,58]. These traits reflect a “slow” or resource-conservative end of the global plant economics spectrum [59,60]. However, some studies indicate that grazing can promote traits associated with fast regrowth (e.g., high specific leaf area) in less arid systems, showing context-dependent divergence between drought and grazing effects [61]. Grazing has previously been found to enhance functional diversity at arid sites but decrease functional diversity at more mesic sites [36]. Similarly, community-weighted means (CWMs) (Table 1) of SLA and LNC increased in response to grazing at arid sites but decreased in response to grazing at mesic sites [53]. These results reflect a complex interaction between herbivory and climate along the fast–slow plant economics continuum, where acquisitive strategies may confer rapid growth in favorable conditions but also increase vulnerability to herbivory and drought stress. Given this background, we expect there to be an interaction between climate and herbivore assemblage on functional trait and functional diversity responses. However, the studies that have investigated the effect of herbivores and climate together on plant functional traits manipulate all herbivores solely via presence/absence rather than separating the impacts of wild herbivores and livestock [62,63], leaving open the question of whether herbivore identity alters these outcomes along the fast–slow spectrum.

Not only do climate and herbivory alter plant functional traits and functional diversity within communities; they also alter variability in traits within species (intraspecific trait variation; ITV; Table 1) [64,65]. However, not all species respond with the same level of variability with regard to climate gradients [66], and not all traits show the same level of plasticity [67]. For example, SLA shows more variability within species than LDMC [68], and the percent of species that respond to grazing with a higher ITV of SLA has been found to increase along a precipitation gradient [64]. The role of ITV in moderating the effects of climate and herbivory is critical to understand because ITV has been found to affect ecosystem processes [69] and affects the response of CWMs and functional diversity to disturbance and environmental change [53,70,71].

Climate and herbivory are ubiquitous drivers that shape terrestrial ecosystems, and the impact both have on ecosystems is changing due to anthropogenic effects. As such, conducting empirical studies in an experimental system that not only manipulates herbivore density and identity in realistic patterns, but is replicated across a climatic gradient, is essential to further piece together the impacts of herbivory on ecosystems in a changing world. By understanding how and when plant functional traits and functional diversity change with different climates and herbivore assemblages, we can better understand how anthropogenic change may alter ecosystem function.

In this study, we experimentally manipulated herbivore composition using exclosure plots to examine the effects of three common forms of herbivore communities in the Anthropocene—total wildlife loss or removal (no herbivores), wildlife without any livestock (wildlife only), and wildlife with cattle (all herbivores) (all hereafter referred to as “treatments”)—on the composition and diversity of plant functional traits at three topo-climatic conditions—arid, intermediate, and mesic—(hereafter referred to as “climate” sites) across an oak savanna ecosystem. Previous research at this location has shown strong changes in plant diversity and cover which change interactively with herbivore assemblage and climate, but to date, no scholar has explored the effects on plant functional traits [45]. Using five commonly examined functional traits (leaf area—LA; specific leaf area—SLA; leaf dry matter content—LDMC; leaf nitrogen concentration—LNC; and seed mass; Table 1) that are known to be linked to various metrics of ecosystem function, we ask the following questions:

(1) How do climate, herbivore assemblage, and their interaction affect plant functional trait composition within a site? We predict that with increasing access by herbivores (from no herbivores to wildlife only to all herbivores), plant communities will have higher values in community-weighted means (CWMs) of LA, SLA, and LNC as well as lower values in LDMC and seed mass due to an increasing abundance of herbivore-tolerant plant species. We expect that these effects would be stronger in more arid climates because species adapted to high levels of aridity have a lower capacity for regrowth after biomass removal (low tolerance) [72], and because, as detailed earlier, aridity and grazing produce synergistic effects [73,74,75].

(2) How much are community traits influenced by species turnover and intraspecific trait variation (ITV)? We expect that CWMs will be influenced by both species turnover and ITV, but we do not know the relative contribution of each to community-level trait variation. We predict the effects of climate will be predominately explained by species turnover, while the effects of herbivore treatment within the same climate will be primarily driven by ITV because we hypothesize aridity will act as a stronger environmental filter than herbivore assemblage. Importantly, we interpret ITV in this context as being largely driven by **phenotypic plasticity** rather than ontogenetic or demographic variation. Herbivore pressure and resource availability may interact to influence plastic trait responses within species particularly for traits like LNC that are known to vary with leaf age and plant developmental stage [59,76]. While we do not explicitly partition sources of ITV in this study, plasticity likely plays a central role in mediating trait shifts in response to herbivore assemblage, and future studies should further explore this mechanism.

(3) How do climate, herbivore assemblage, and their interaction affect plant functional diversity within a community? We predict that increases in aridity (a major stressor) may lead to reduced functional diversity (lower values of functional richness [FRic], functional divergence [FDiv], functional evenness [FEve], and functional dispersion [FDis]; Table 1). Additionally, we predict that a larger effect will be observed with increased access to grazing (no herbivores to all herbivores) if aridity and herbivore presence act as filters and cause species to converge on specific trait values. Conversely, herbivory may increase functional diversity if grazing acts to release plants from a dominant competitor [4].

## 2. Results

### 2.1. Community-Level Changes in Functional Traits

As predicted, climate interacted with herbivore treatment to influence LA_CWM_ (Table S6). The removal of all large herbivores had a positive effect on LA_CWM_ at arid (*p* < 0.001), a neutral effect at intermediate, and a negative effect at mesic (*p* < 0.01). The removal of only cattle (wildlife only treatment) had a neutral effect on LA_CWM_ at arid and mesic, and it had a positive effect at intermediate (*p* = 0.003). LA_CWM_ increased with aridity but only at the no herbivore treatments (Arid–Int: *p* = 0.0001, Int–Mesic: *p* < 0.01, Mesic–Arid: *p* < 0.0001). Climate had no effect on wildlife only treatments. Between all herbivore treatments, LA_CWM_ was larger at mesic than intermediate (*p* < 0.01).

Similarly, climate interacted with herbivore treatment to influence SLA_CWM_ (Table S6). The wildlife only treatment (as compared to all herbivore treatment) had no significant effect on SLA_CWM_. The no herbivore treatment (as compared to all herbivore treatment) increased SLA_CWM_ but only at arid (*p* < 0.0001). Similar to LA_CWM_, we see a step-wise increase in SLA_CWM_ with aridity but only in the no herbivore treatment (Arid–Int: *p* < 0.01, Int–Mesic: *p* = 0.01, Mesic–Arid: *p* < 0.0001). A similar, non-significant trend was observed within all herbivore treatments. Within wildlife only treatments, intermediate was larger than both arid (*p* = 0.048) and mesic (*p* = 0.01).

Only herbivore treatment was present in the model for LDMC_CWM_ (Table S6). All herbivore treatments showed larger LDMC_CWM_ values than wildlife only treatments (*p* < 0.01), but the difference was not significant between other treatment types.

**Figure 1:**
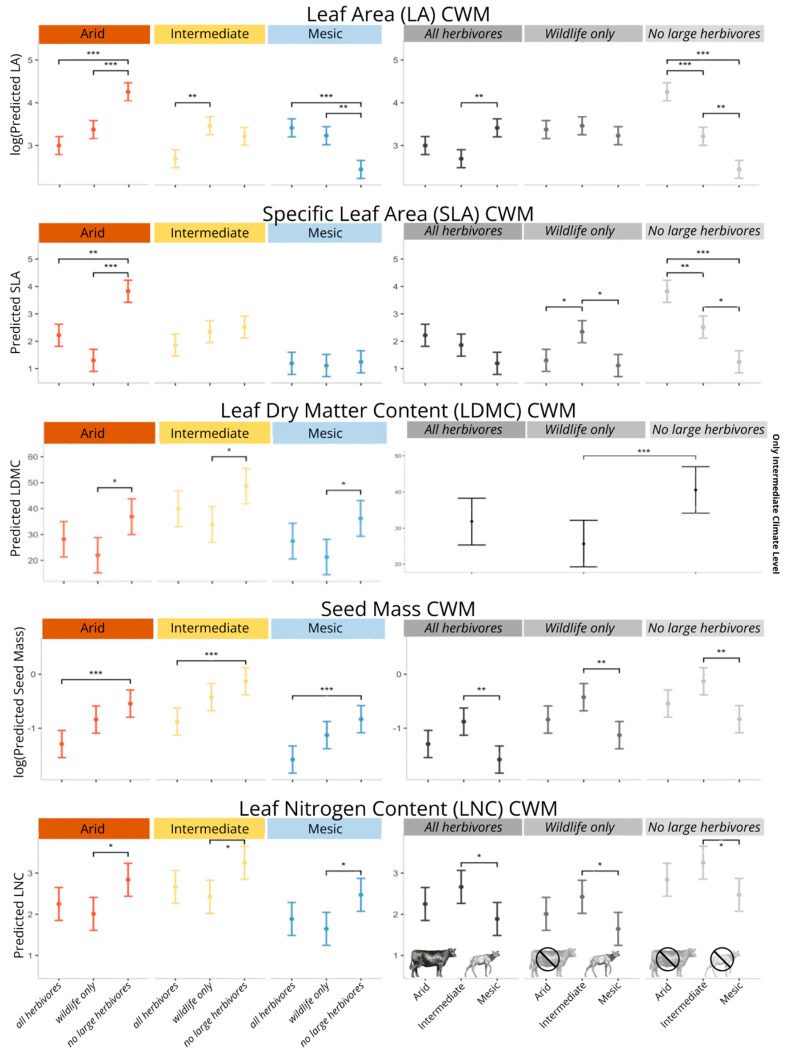
Estimate marginal means of community-weighted means (CWMs) by climate and herbivore treatments. The first column shows relationships by climate, and the second column shows relationships by herbivore treatment. Significance is denoted as follows: *p* < 0.05 (*), *p* < 0.01 (**), *p* < 0.001 (***).

Both climate and treatment had important influences on seed mass_CWM_ and LNC_CWM_, but treatment effects did not vary by climate for these metrics (Table S6). For all climates, seed mass_CWM_ was larger in no herbivore sites than all herbivore sites (*p* < 0.001) (Figure 1). For all herbivore treatments, seed mass_CWM_ values were larger at intermediate than mesic (*p* < 0.01). Between herbivore treatments, LNC_CWM_ values were larger in no herbivore treatments than wildlife only treatments at all climates (*p* = 0.03). Across herbivore treatments, LNC_CWM_ was higher in intermediate than mesic (*p* = 0.048) (Figure 1).

### 2.2. Species Turnover and ITV

For all traits, both species turnover and intraspecific trait variation (ITV) accounted for the sources of variation within the community (Table 2 and Table S10), but the relative contribution of each differed between the measured traits. SLA_CWM_ and LDMC_CWM_ variation were explained predominately by species turnover (79% and 76% of total variation, respectively), while LA_CWM_ variation was more evenly influenced by both species turnover and ITV. Species turnover and ITV positively covaried for total sources of variation. For SLA_CWM_, species turnover and ITV were positively correlated for all sources of variation (Table 2). Only for variation due to climate treatment for LA_CWM_ and variation due to climate and herbivory interaction for LDMC_CWM_ did we see any negative covariation in experimental treatments (Figure 1).

### 2.3. Functional Diversity

Metrics of functional diversity responded differently to climate and grazing treatment, and responses were not consistent across metrics. Best-fit models for FDiv and FDis included climate, treatment, and their interaction (Tables S8 and S9). However, neither climate or herbivore treatment significantly altered FEve and FRic with the models including the intercept only.

**Figure 2:**
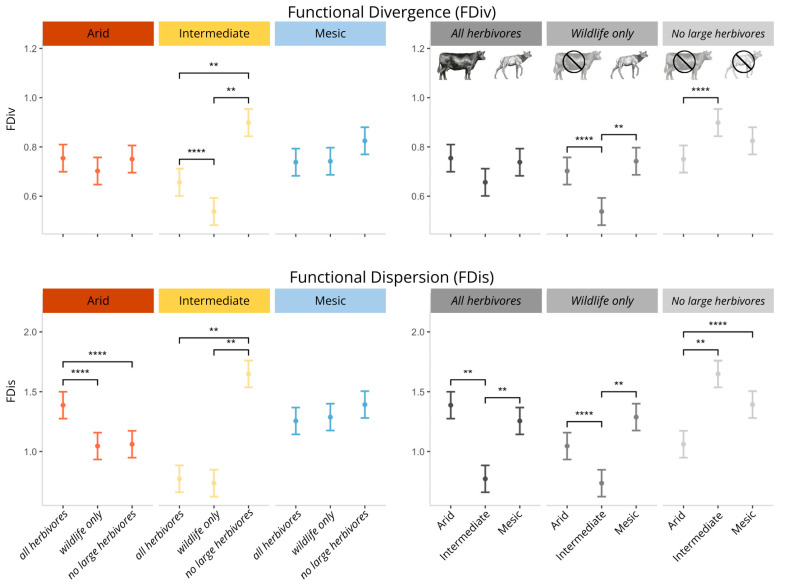
Estimate marginal means for functional diversity metrics functional divergence and functional dispersion (FDiv and FDis) by climate and herbivore treatment. Significance is denoted as follows: *p* < 0.01 (**), *p* < 0.0001 (****).

For FDiv, only the intermediate climate experienced an effect of herbivore treatment with the removal of cattle (wildlife only) causing a decrease in FDiv (*p* = 0.02), and the no herbivore treatment causing an increase (*p* ≤ 0.0001) as compared to all herbivore treatments (Figure 2). Within the no herbivore treatment, FDiv was larger at arid than intermediate (*p* < 0.01) (Figure 2). Within the wildlife only treatments, FDiv was larger at both mesic and arid than intermediate (*p* < 0.01, *p* = 0.02, respectively). There were no differences in FDiv between all herbivore treatments across the climates (Figure 2).

For FDis, values were significantly larger at mesic than both arid (*p* = 0.02) and intermediate (*p* = 0.0001) when averaged over treatment. There was no effect of herbivore treatment in the mesic climate. At intermediate, no herbivore treatments had higher FDis than in either all herbivore (*p* ≤ 0.0001) or wildlife only plots (*p* ≤ 0.0001). At arid, all herbivore plots had higher FDis than either wildlife only (*p* = 0.01) or no large herbivore plots (*p* = 0.02). Within both all herbivore treatments and wildlife only treatments, FDis was larger in mesic and arid than intermediate (*p* = 0.0001, *p* = 0.03, respectively) with no difference between arid and intermediate. For no herbivore treatment plots, FDis values were significantly smaller at arid than both mesic and intermediate (*p* = 0.02, *p* = 0.0001, respectively) with no difference between mesic and intermediate (Figure 2).

## 3. Discussion

Large herbivores have numerous important ecological effects on herbaceous plant communities [1,2,3]. Alarmingly, herbivore assemblages are rapidly altering worldwide [4,5,6], alongside the growing pressures of climate change, which also has been well documented to have strong effects on plant communities [77,78]. When designing this study, the climate gradient between arid, intermediate, and mesic sites was intended to simulate the pressure of climate change—specifically the increase in hotter and drier climates in this region—with the study sites varying by ~2.5 °C approximating a low to intermediate emissions warming scenario [79]. Combined with the herbivore manipulations, this allowed us to understand how realistic changes in large herbivore identity and presence would likely influence plant functional traits and functional diversity under changing climatic conditions. Our results showed that both climate and herbivore treatment, as would be expected, impacted plant functional traits and functional diversity. More revealingly, we also frequently observed significant interactions between climate and herbivore treatment. Notably, climatic context sometimes changed not only the magnitude but also the direction of the responses, even within this spatially constrained oak savanna ecosystem. Equally critically, we often detected differences in effects across the types of herbivore treatment; for some responses, the effects of fencing seemed to roughly track herbivore abundance, while for other responses, herbivore identity seemed to matter more than herbivore abundance. Here again, the pattern of effects also often differed across climatic contexts. While this matched our most general predictions (that there would be climate by herbivore interactions, and differences by herbivore assemblage), the specifics of functional trait responses did not always meet our predictions. These differences were likely due to differences in plant community similarity, changes in the dominance of plant functional groups, and the differing ecology of herbivore communities across sites.

### 3.1. The Response of Community-Level Trait Weighted Means to Grazing and Climate

We observed differing effects of climate on LA_CWM_ and SLA_CWM_ across herbivore treatments. In the absence of large herbivores (no herbivore treatment), LA_CWM_ and SLA_CWM_ increased in a stepwise fashion, from lower values at mesic climate sites to higher values at arid climate sites, indicating the presence of drought-tolerant species in arid sites compared to more drought-avoidant species at mesic sites [51,54].

Looking instead at the effects of herbivores within a climate level, we find that contrary to other studies and our predictions, at arid sites, LA_CWM_ and SLA_CWM_ increased from all herbivore and wildlife only treatments (which were statistically indistinguishable) to no herbivore treatments, suggesting that in arid contexts, only livestock affected these traits [22,53]. In mesic climatic contexts, it was again the addition of livestock that caused changes in LA_CWM_, but in this case LA_CWM,_ increased when livestock were added, which is more consistent with theory [22,53], and there were no differences in SLA_CWM_ in mesic sites. It should be noted that while these effects appear to be largely associated with the addition of cattle, and not with increasing herbivore density, we lacked a livestock-only treatment due to feasibility. Thus, we were unable to fully explore the reasons that livestock addition caused these effects. As livestock are additive to wildlife in this system, it could be that the emergence of these effects only in all herbivore plots was due to higher total grazing intensity, or it could be due to the differing effects of livestock as grazers, as different sized herbivores are known to uniquely shape functional trait composition [43]. The level of grazing intensity can impact the types of effects observed [3,30,80]. At low-intensity grazing, herbivores remove large leaves, but not to the degree that would increase the abundance of herbivore-tolerant species, so it effectively decreases LA and SLA.

The reasons for different plant trait responses to herbivores across climatic contexts may be driven by plant community shifts between herbivore avoidance and herbivore tolerance strategies along our climate gradient [3,28,29]. Specifically, the higher values of LA_CWM_ and SLA_CWM_ with herbivore removal (no herbivore treatment) at arid suggest that the plant species with herbivore avoidance strategies increase at high herbivore densities/when cattle are present. However, the decrease in LA_CWM_ within no herbivore plots at mesic suggests an herbivore tolerance strategy in the presence of cattle. Overall, we do not seem to observe an additive effect of climate and herbivore assemblage as predicted.

Interestingly, when herbivores were present, there were no systematic increases in LA_CWM_ and SLA_CWM_ from mesic to arid climate sites, such as the stepwise trend we saw in no herbivore treatments. Instead, there were even occasional decreases in LA_CWM_ and SLA_CWM_ from mesic to arid climate sites. This difference in plant responses to herbivore assemblage between climates may also be due to differences in plant community dominance. A study at the site by Orr et al. (2022) [45] found that while the plant community at arid was dominated by invasive annual grasses, intermediate was dominated by both this invasive annual grass and a native woody shrub, and mesic was dominated by a native shrub and a native annual forb. Combined with our findings of high community dissimilarity between herbivore treatments at arid (Section 4), the differences in LA_CWM_ and SLA_CWM_ at arid may be led by an increase in abundance by invasive annual grasses, which have previously been found to increase in abundance in arid for both wildlife only and no herbivore treatments [45]. Such grasses are known to have both drought-tolerant and herbivore-tolerant traits, which would explain the high values of LA_CWM_ and SLA_CWM_ we found in the no herbivore treatments, as these strategies both tend to have higher LA and SLA associated with them [48,51].

### 3.2. The Contribution of ITV and Species Turnover to Community-Level Traits

Species turnover accounted for the majority of trait variability due to climate and treatment effects for SLA_CWM_ and LDMC_CWM_ and almost half the variability of LA_CWM_. This follows our prediction for the high contribution of species turnover to trait variability due to climate, and it is consistent with other research that has shown that the relative contribution of species turnover increases across environmental gradients and with environmental harshness [65,81]; however, it does not follow our prediction that intraspecific trait variation (ITV) would explain more variability for herbivore treatment. ITV accounted for a relatively small amount of the response of SLA_CWM_ and LDMC_CWM_ variability to climate, treatment, and their interactions, which may be indicative of how leaf-level traits show less ITV than plant-level traits [82]. However, this does not hold true for the almost equal contributions of species turnover and ITV we observed for LA_CWM_.

The positive correlation we observed between species turnover and ITV when explaining total variation suggests that climate and herbivory are changing community functional traits by both selecting for species with specific functional traits and by altering within-species trait values in a way that reinforces those community trait responses. However, it should be noted that we had high levels of dissimilarity in understory species between our climate treatments, making it more difficult to interpret the relative contributions with respect to climate using this methodology.

### 3.3. The Response of Functional Diversity to Climate and Herbivory

We found significant effects of both climate and herbivore assemblage on FDis and FDiv, and differences between climate and treatments appear to be driven by differences in functional group dominance (as observed by Orr et al., (2022) [45]) and herbivore identity. Livestock addition appears to be driving FDis patterns at arid, whereas wild herbivores appear to be driving patterns at intermediate. FDis values were higher overall at mesic climate than both intermediate and arid, meaning that species’ traits are less centered around the community trait mean, which indicates that species’ trait values, as well as species abundances, are more even with increasing aridity at intermediate and arid. This makes sense, as increasing aridity imposes abiotic stressors that act as environmental filters [18].

The difference in FDiv values between herbivore treatments at intermediate indicates that when all herbivores were removed, there was a higher abundance of species with trait values that diverged from one another. This divergence may be due to the high abundance of both annual grasses and native shrubs at the intermediate climate, as studies on the effects of herbivores on grasses and shrubs often have opposite results [83].

Surprisingly, there was also no effect of climate on FEve and FRic, even though we see a significant difference in climate conditions, high community dissimilarity between climates, and a change in dominant species. In particular, FEve includes species’ abundances in its calculation, so one might expect it to be more sensitive to changes in plant community composition. However, studies have found that functional diversity metrics respond differently than species diversity to environmental stress and change [84]. While functional diversity metrics may not align with predictions based on species diversity [37], they can be better indicators of community assembly than species richness [85]. While both climate and herbivore assemblage were found to significantly alter plant species diversity at this site [45], our findings show that this does not reduce functional richness or evenness, indicating that some aspects of ecosystem function may be conserved even with large changes in species between treatments.

### 3.4. Broader Implications

In this study, we used a multi-factor large herbivore exclosure experiment to simulate three of the most common large herbivore change scenarios globally (defaunation, conserved wildlife-only, and livestock addition) and explored the interaction between increasing aridity and altering herbivore community composition on the functional trait composition of herbaceous plant communities in an oak savanna ecosystem. We found that even over a relatively large climate gradient for this landscape, the effects of herbivores on community-level traits can be very large, sometimes even exceeding that of climate change alone.

Moreover, our study found an interactive effect between changes in large herbivore grazing and climate on community-level functional traits with climate context sometimes inverting the community-level response to grazing. We determined this change was due to shifts from herbivore tolerance to avoidance traits, which were particularly reflected by changes in plant leaf area and specific leaf area. Our study additionally demonstrates that livestock and large wild herbivores can differ in the magnitude and direction of effects on functional traits and diversity; however, we were unable to fully separate the extent to which these effects were driven by the density vs. identity of herbivores across herbivore treatments. Further, those responses can be reversed or halted via interactions with climate with climate often only affecting trait responses due to the additional removal of wild herbivores but not cattle-only removal. Cumulatively, these findings highlight the importance of herbivore management in mitigating and adapting to climate change.

As humans continue to alter ecosystems, it is becoming increasingly important to understand the resulting effects on functional traits and functional diversity as they are inextricably tied with ecosystem functioning. This is particularly true for California Mediterranean ecosystems and oak savannas which are under threat from climate change [86] and are extensively grazed by livestock. Through this study, we highlight the challenges of generalizing the effects of either herbivore communities or climate on complex responses such as functional trait and diversity responses. By understanding the interaction between climate and herbivore assemblages, we are better able to predict how critical functional traits will be altered in the future.

Our finding of an interaction effect between grazing and climate on community-level functional traits illustrates the importance of considering the role of climate context and herbivore assemblage together when predicting functional trait changes. This highlights the importance of rangeland management in vegetation adaptation to climate change. Therefore, we may be able to utilize grazing to maintain community-level traits important to ecosystem function, potentially mitigating the effects of climate change to some degree. While this may be most easily accomplished through the use of livestock, it also would be applicable to sites where the rewilding and recovery of native herbivores may be possible.

## 4. Materials and Methods

### 4.1. Study Site

**Figure 3:**
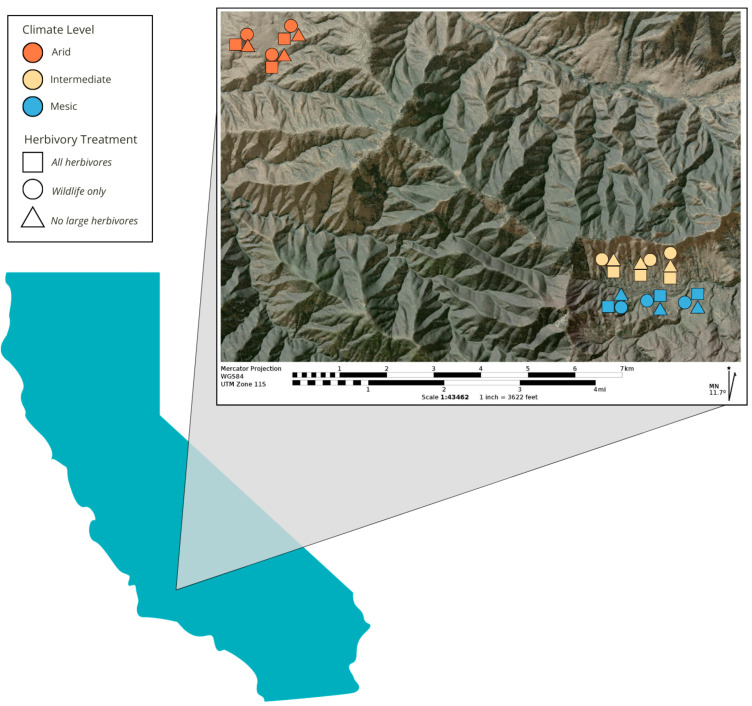
Location of the Tejon Ranch Exclosure Experiment (TREE) in south–central California, and the approximate location of the 27, 1 hectare plots across three climate and three herbivore treatments (not drawn to scale). Realistically on the landscape, all of the 1 hectare plots are square, but with this figure, to distinguish between the herbivory treatment types, the plots that are square are all herbivore plots, circles are wildlife only plots, and triangles are no large herbivore plots. These plot icons are colored by which climate level they are located: dark orange for the arid climate, golden yellow for the intermediate climate, and light blue for the mesic climate level.

We conducted this study at the Tejon Ranch Exclosure Experiment (TREE) located at Tejon Ranch in Kern Co., Lebec, CA, USA (34°5′80″ N, 118°3′50″ W). Established in 2016, TREE consists of 27, 1 hectare plots which include three replicates (blocks) of three herbivore treatments at each of three topo-climate conditions (Figure 3). The treatments used barbed-wire fenced plots to simulate some of the common forms of herbivore compositional change globally and regionally: (1) no large herbivores (no herbivores, simulating defaunated lands), (2) wildlife only present (cattle-only removed, simulating conserved lands not grazed by livestock), and (3) unfenced plots (plot corners demarcated with t-posts) with cattle and wildlife present (all herbivores, indicative of managed grazing lands). Livestock only treatments were not feasible given that livestock in this system are free roaming and there was no fence design that would exclude wildlife only; but livestock only is also not a common form of land use in these open pasturelands, such that this did not seem to be a major omission from a management perspective.

These treatments are repeated across three aridity/climate types: mesic (high water availability), intermediate, and arid (low water availability). To understand each site’s community composition, we calculated a Jaccard dissimilarity index using the community abundance data for the species constituting 90% cover in each plot. To calculate significant differences between plots and climates, we performed three analyses of similarities (ANOSIM), one investigating the effect of climate on plant community and one for each climate treatment to compare the effect of herbivore treatment within each climate. The analysis returns an *R* statistic where a value of 1 indicates complete dissimilarity.

We found that between climate treatments, there was a very high level of dissimilarity (*R* = 0.9646, *p* = 0.001). Within climates, arid had a high amount of dissimilarity (*R* = 0.8436, *p* = 0.008), which was followed by mesic (0.7284, *p* = 0.016), and finally, intermediate (*R* = 0.3086, *p* = 0.029). Details on the site’s three climate types are found in Tables S1–S3 and have been described in detail in [45]. Vegetation across the sites consists of oak savanna, with the predominant canopy consisting of blue oak (*Quercus douglasii*), valley oak (*Q. lobata*), and black oak (*Q. kellogii*), with density and species of oak varying with climate type. The majority of the understory consists of non-native grasses (*Bromus diandrus, B. hordeacous*) in *arid* sites, non-native grass (*B. diandrus*) and native woody shrubs (*Ericameria nauseosa*) in intermediate sites, and native woody shrubs (Ribes californicum var. hesperium) in mesic sites. Soils are loamy granite residuum Haploxerolls across all sites [87]. Mesic sites are sloped north facing, intermediate sites are sloped south facing, and arid sites have minimal slope. Large herbivores common in the region consist of mule deer (*Odocoileus hemionus*), introduced Rocky Mountain elk (*Cervus elaphus nelsoni*), invasive wild boar (*Sus scrofa*), and free-ranging cattle (*Bos taurus*).

This experiment provides an ideal scenario for detecting the effects of realistic herbivore change by controlling the access of cattle and wild herbivores across climate contexts, allowing us to investigate herbivore assemblage interactions with climate on plant communities. Notably, the climate treatments of this experiment were selected to represent three probable future climate change scenarios that oak savannas will experience in California [45].

### 4.2. Herbivore Community

**Figure 4:**
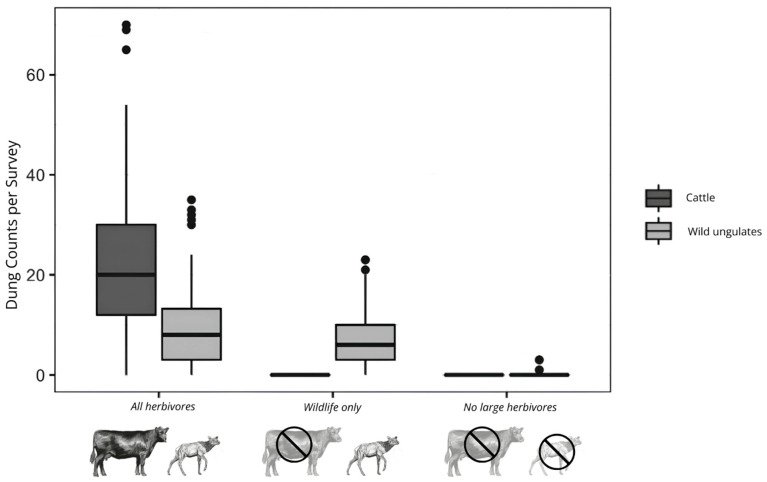
Dung density by herbivore treatment (from Orr et al., 2022 [45]). Total wild ungulate dung counts did not differ between all herbivores and wildlife only treatments. Upper and lower limits of the box represent the first and third quartiles, lines represent 1.5 times the interquartile range, and points represent outliers.

Dung counts conducted within each plot throughout 2017 and 2018 were used to determine herbivore density and composition [45]. These data show that herbivore treatments successfully reduced total herbivore densities and that those densities did not differ significantly between climate levels. Critically, total densities of wild herbivores were not affected by the wildlife only treatment, and the no large herbivore treatments effectively excluded all large herbivores. Therefore, impacts from cattle can be considered additive (Figure 4).

### 4.3. Plant Functional Trait Collection

In order to determine how herbivore and climate alter plant functional traits, we chose a suite of easy-to-measure functional traits that are indicative of how both biotic and abiotic change impact ecosystem function: leaf area (LA), specific leaf area (SLA), leaf dry matter content (LDMC), leaf nitrogen concentration (LNC), and seed mass. Plant traits were collected in Spring 2017 for common species at one plot of each herbivore treatment type (all herbivores, wildlife only, no large herbivores) per each aridity level (*n* = 9). Common species included those comprising at least 90% of the total understory plant cover in each plot based on spring transect vegetation surveys (Table S4). Species abundance was determined from vegetation surveys conducted in Spring 2019 within two weeks of each site’s peak Normalized Difference Vegetation Index (NDVI). Methods are detailed in Orr et al. (2022) [45], but briefly, a 1 m^2^ quadrat was placed at 10 m intervals along six 50 m transects in each plot (36 m^2^ per plot) to determine species cover (up to 100% for each species).

The collection of functional traits for community trait analysis followed the methods of Cornelissen et al. (2003) [67]. Specifically, two mature leaves were collected from ten individuals of each common species, and when possible, from full-sun areas. Effort was made to spread leaf collection throughout the whole plot, with a minimum sampling distance of five meters between individuals, unless there were fewer than ten individuals located within the entire plot. Plant samples were kept in a cooler box on ice until they could be placed into beakers of water and kept in a large refrigerator (4 °C) for up to 48 h before processing.

Leaf area was determined by scanning leaves with a digital scanner and using ImageJ v1.53k [88], which computed each leaf’s area. Then, LA and fresh and dried leaf weights were calculated in order to determine SLA and LDMC. Leaf samples were weighed fresh (after being wiped to remove any residual moisture), dried at 60 °C for at least 36 h, and weighed again for dry weight.

LNC and seed mass values were obtained from the TRY Plant Trait Database [89]. Any missing trait values were substituted with additional trait collection in 2019, or by using closely related species, genus-level or family-level records (substitutions outlined in Table S5).

### 4.4. Community-Level Trait Analysis

We performed a community-level analysis to understand how the community expressions of traits were influenced by climate and herbivore assemblages, calculating the community-weighted mean (CWM) for each functional trait in each plot. These were calculated by taking the mean of each species’ trait value at a plot weighted by their 2019 cover abundance ($CWM=\overset{n}{\underset{i}{\sum }}t_{i}\times a_{i}$; where *n* = number of species in each plot, *t_i_* = trait value for *i*th species, and *a_i_* = abundance for the *i*th species at a plot).

### 4.5. Plant Functional Diversity

To capture the overall functional diversity, we calculated functional richness (FRic; the convex hull, or volume, of functional trait space [90]). To capture variation in functional diversity, we calculated functional divergence (FDiv; species divergence from the mean [90]), functional evenness (FEve; the regularity of abundances of each species within the functional space [90]), and functional dispersion (FDis; the average distance of species to centroid weighted by their abundance [91]). Each metric was calculated using the FD package in R (v1.0-12 [92]).

### 4.6. Modeling of Plant Functional Traits and Plant Functional Diversity

To determine the best predictors of variation between the CWMs of the five plant functional traits and the functional diversity responses of the plant community across herbivore and climate treatments, we built linear mixed effects models (LMMs) using the glmmTMB R package (v1.0.1 [93]). We leveraged combinations of climate, herbivore treatment, and their interaction as fixed effects and the specific plot location (a categorical variable represented by letters A-I) as a random effect. We selected for best-fit models by minimizing Akaike information criterion adjusted for small sample sizes (AICc) values using the MuMIn R package (v1.43.17 [94]). If AICc of multiple models were within two units, we chose the model with the fewest parameters [95]. We verified that model assumptions were met by visually inspecting the normality of residuals using the simulateResiduals function from the DHARMa R package (v0.4.1 [96]). Using the best-fitting LMM, we examined pairwise differences using the “emmeans” function as a post hoc test (“emmeans” R package, v1.6.0 [97]) A log(x) transformation was performed on the CWMs of LA and seed mass in order to meet model assumptions about normality and variance.

### 4.7. Species Turnover and ITV

To determine the relative contributions of species turnover and intraspecific trait variation (ITV) to community functional trait changes, we decomposed the variation in CWM values into variability from intraspecific trait variability, variability from species turnover, and their covariation utilizing methods from Lepš et al. (2011) [98], including the R function trait.flex.anova (see [98]). Following their methods, we utilized three, two-way analysis of variance (ANOVA), or permutational analysis of variance (PERMANOVA) for non-normal data, one using “specific” averages, one using “fixed averages”, and one using ITV values. “Specific” averages weigh a species’ site-specific values by their abundances and are used to represent total variation. Therefore, we used our previously calculated CWMs as the response variable. “Fixed” averages weigh species’ abundances by an average, or fixed, trait value from all the locations where this species occurs and are used to represent species turnover. Therefore, we calculated the average trait value for each species from every plot where they occurred as the response variable. To calculate ITV, we subtracted our fixed averages from our specific averages. All ANOVAS and PERMANOVAS took the following form: response variable~climate × treatment.

After performing these three ANOVAS for each of our traits, we used their total sum of squares (SS) to determine the relative contribution of species turnover and ITV to the trait variability by decomposing the amount of variability explained by climate, treatment, and their interactions and unexplained error (SS_total_ = SS_climate_ + SS_treatment_ + SS_treatment:climate_ + SS_error_). Further following Lepš et al. (2011) [98], we expect that SS_specific_ = SS_fixed_ + SS_ITV_ with species turnover and ITV effects varying independently. By observing whether SS_specific_ is lower than expected, we can then look to see if SS_fixed_ and SS_ITV_ are positively or negatively correlated. If positively correlated, then SS_specific_ will be higher than expected, meaning they are selecting for the same trait values. When negatively correlated, SS_specific_ will be lower than expected, and they are selecting for conflicting trait values. Lepš et al. expands on this further by determining their covariations as CovSS = SS_specific_ − SS_fixed_ − SS_ITV_, where positive and negative CovSS values mean a positive and negative covariation, respectively. We used this method, and the trait.flex.anova, to perform the three ANOVAS for LA, SLA, and LDMC and decompose their variances. We did not evaluate seed mass or LNC due to a lack of site-specific trait values.

## Figures and Tables

**Table 1:** Abbreviations and definitions of plant and plant-community functional traits.

Term	Abbreviation	Definition
Intraspecific Trait Variation	ITV	Variation in trait values between member of the same plant species
Community-Weighted Mean	CWM	Community means of each species’ trait value weighted by their plot-specific abundance
Leaf Area	LA	One-sided leaf area (mm^2^)
Specific Leaf Area	SLA	Leaf area/dry mass (mm^2^ × (mg^−1^))
Leaf Dry Matter Content	LDMC	Leaf dray mass/fresh leaf mass (mg × (g^−1^))
Seed Mass		Weight of dry seed (mg)
Leaf Nitrogen Content	LNC	Total leaf nitrogen content per dry mass of leaf matter (mg × (g^−1^))
Functional Richness	FRic	The convex hull, or volume, of a plant community’s functional trait space
Functional Divergence	FDiv	Distance of abundance-weighted trait values from the center of the communities’ functional space
Functional Evenness	FEve	The regularity of abundances of each species within the functional space
Functional Dispersion	FDis	The average distance of species to centroid weighted by their abundance

**Table 2:** Relative contributions (%) of species turnover and intraspecific variation (ITV), their covariation, and the total variation for when a significant result of climate level, herbivory treatment, or an interaction on plant functional traits was found.

CWM	Terms	Species Turnover	ITV	Covariation	Total
Leaf Area (LA)	Climate	6.11	18.29	−4.75	19.65
	Treatment	3.74	1.96	5.09	10.79
	Climate:Treatment	18.33	15.58	19.18	53.09
	Residuals	14.87	2.97	−1.36	16.47
	Total	43.05	38.79	18.15	100.00
Specific Leaf Area (SLA)	Climate	33.08	2.17	1.91	37.16
	Treatment	15.86	0.57	4.01	20.44
	Climate:Treatment	16.29	2.98	7.61	26.88
	Residuals	14.13	0.26	1.13	15.52
	Total	79.37	5.98	14.65	100.00
Leaf Dry Matter Content (LDMC)	Climate	17.01	0.79	6.70	24.51
	Treatment	16.10	1.94	9.67	27.71
	Climate:Treatment	16.23	0.35	−0.48	16.11
	Residuals	27.02	0.59	4.06	31.67
	Total	76.37	3.68	19.96	100.00

## Data Availability

The dataset and code are available at https://knb.ecoinformatics.org/view/doi:10.5063/F1SQ8XWP (accessed on 14 April 2025).

## Supplementary Materials

The following supporting information can be downloaded at https://www.mdpi.com/article/10.3390/plants14081249/s1, Table S1: ANOVA results for climate variables. Table S2: Tukey HSD pairwise comparisons for each climate variable. Table S3: Climate data using 30 m downscaled PRISM data [86,99]. Table S4: 2017 Taxa list and 2019 abundances. Table S5: Additional data sources [89]. Table S6: Community-weighted means (CWMs) linear mixed effect models linear mixed effect models, Akaike information criterion (AIC) and degrees of freedom (df). The fixed effect are herbivory treatment and climate level with a random effect of “plot”, which was one of the twenty-seven treatment locations from where samples were collected (plot location = PL). Final best-fit models are in bold. Table S7: Community-weighted means (CWMs) linear mixed effect models conditional and marginal R2. Table S8: Functional diversity metrics linear mixed effect models, Akaike information criterion (AIC) and degrees of freedom (df). The fixed effects are herbivory treatment and climate level with a random effect of “plot”, which was one of the twenty-seven treatment locations from where samples were collected (plot location = PL). Final best-fit models are in bold. Table S9: Functional diversity metrics linear mixed effect models conditional and marginal R2. Table S10: ANOVA and PERMANOVA tables for functional trait community-weighted means (CWMs) specific averages, fixed averages, and intraspecific trait variation (ITV).
