# Supplementary material for: Interactive Effects of Climate and Large Herbivore Assemblage Drive Plant Functional Traits and Diversity
Source: Plants (Basel). 2025 Apr 20;14(8):1249. doi: 10.3390/plants14081249 (PMC12030475; doi:10.3390/plants14081249)
Supplement: Supplementary file 1 [file plants-14-01249-s001.zip › plants-3557935-supplementary.pdf]

SUPPLEMENT TO:

Interactive Effects of Climate and Large Herbivore Assemblage Drive Plant Functional Traits and Diversity

Running title

Effects of Aridity and Herbivores on Plant Functional Traits

Authors

Maggie Klope[1], Ruby Harris-Gavin[1], Stephanie Copeland[1], Devyn Orr[1], Hillary S Young[1*]

[1]Department of Ecology, Evolution, and Marine Biology, University of California Santa Barbara, Santa Barbara, CA, USA

[*]Corresponding author

hillary.young@lifesci.ucsb.edu

+1 619 889 7520

Climate Treatments

Table S1. *ANOVA results for climate variables.*

Climate Variable	Source of Variation	Degrees of Freedom	Sum of Squares	Mean Square	Iteration	p
Climate Water Deficit	Climate	2	90539.420	45269.709	5000	<.0001
	Treatment	6	42122.630	7020.439	5000	<.0001
	Residuals	18	16265.640	903.647		
Potential Evapotranspiration	Climate	2	75829.430	37914.717	5000	<.0001
	Treatment	6	47301.130	7883.522	5000	0.001
	Residuals	18	20191.600	1121.756		
Precipitation	Climate	2	3123.313	1561.657	5000	<.0001
	Treatment	6	15.834	2.639	732	0.2
	Residuals	18	33.776	1.876		
Mean Daily Maximum Temperature	Climate	2	12.425	6.213	5000	<.0001
	Treatment	6	0.189	0.032	5000	<.0001
	Residuals	18	0.029	0.002		
Mean Daily Minimum Temperature	Climate	2	7.671	3.835	5000	<.0001
	Treatment	6	0.018	0.003	5000	0.02
	Residuals	18	0.012	0.001		

Table S2. *Tukey HSD pairwise comparisons for each climate variable.*

Climate Variable	Comparison	Difference	Lower Bound	Upper Bound	p
Climate Water Deficit	Intermediate–Arid	108.588	72.422	144.755	<.0001
	Mesic–Arid	133.328	97.162	169.494	<.0001
	Mesic–Intermediate	24.739	-11.427	60.905	0.2
Potential Evapotranspiration	Intermediate–Arid	124.247	83.952	164.542	<.0001
	Mesic–Arid	94.684	54.389	134.979	<.0001
	Mesic–Intermediate	-29.563	-69.858	10.732	0.2
Precipitation	Intermediate–Arid	-7.432	-9.080	-5.784	<.0001

	Mesic–Arid	-25.605	-27.253	-23.957	<.0001
	Mesic–Intermediate	-18.173	-19.821	-16.525	<.0001
Mean Daily Maximum Temperature	Intermediate–Arid	0.358	0.310	0.407	<.0001
	Mesic–Arid	1.584	1.536	1.633	<.0001
	Mesic–Intermediate	1.226	1.178	1.274	<.0001
Mean Daily Minimum Temperature	Intermediate–Arid	0.261	0.229	0.292	<.0001
	Mesic–Arid	1.238	1.207	1.269	<.0001
	Mesic–Intermediate	0.978	0.946	1.009	<.0001

Table S3. *Climate data using 30 m downscaled PRISM data (Davis, 2018; McCullough et al., 2016).*

**Climate Level**	**Elevation (m)**	**Total Annual Precipitation (mm)**	**Mean Daily Minimum Temperature (°C)**	**Mean Daily Maximum Temperature (°C)**	**Climate Water Deficit (CWD)**	**Potential Evapotranspiration (PET)**
Arid	848.78	366.58	9.26	21.96	1155.95	1515.61
Intermediate	1581.78	428.28	6.23	17.92	1002.37	1483.80
Mesic	1668.11	431.94	6.25	17.99	882.10	1303.40

We used PRISM climate data downscaled to 30 meters (McCullough et al., 2016) to determine if our climate sites differed significantly in total annual precipitation (ppt), mean daily maximum temperature (tmin), mean daily minimum temperature (tmax), climate water deficit (CWD) and potential evapotranspiration (PET). We averaged the data for 1983–2013 and calculated each climate metric for our 27 plots. Due to non-normality and/or unequal variances, we performed PERMANOVAS using each variable as a function of our climate treatments with herbivore treatment replicates as a blocking factor. We then performed Tukey's HSD to determine the significance between climate treatments. All climate treatments significantly differed in ppt, tmax, and tmn (Tables S1–2). Mesic and intermediate did not significantly differ in CWD or PET (Table S1-2).

Species lists, abundances, and trait data sources

Table S4. *2017 taxa list and 2019 abundances.*

Climate	Treatment	Taxa	Abundance %
Arid	*All Herbivores*	*Bromus hordeaceus*	27.46
Arid	*All Herbivores*	*Erodium* sp.	25.73
Arid	*All Herbivores*	*Medicago polymorpha*	8.15
Arid	*All Herbivores*	*Bromus diandrus*	6.37
Arid	*All Herbivores*	*Acmispon wrangelianus*	5.02
Arid	*All Herbivores*	*Trifolium* sp.	4.81
Arid	*All Herbivores*	*Triteleia ixioides*	2.84
Arid	*All Herbivores*	*Gilia tricolor*	2.76
Arid	*All Herbivores*	*Plagiobothrys nothofulvus*	2.47
Arid	*All Herbivores*	*Hordeum murinum*	1.93
Arid	*All Herbivores*	Cerastium glomeratum	1.77
Arid	*All Herbivores*	*Leptosiphon* sp.	1.46
Arid	*Wildlife Only*	*Bromus diandrus*	32.98
Arid	*Wildlife Only*	*Bromus hordeaceus*	13.39
Arid	*Wildlife Only*	*Trifolium* sp.	5.89
Arid	*Wildlife Only*	*Bromus tectorum*	4.63
Arid	*Wildlife Only*	*Erodium* sp.	4.46
Arid	*Wildlife Only*	*Hordeum murinum*	4.38
Arid	*Wildlife Only*	*Triteleia ixioides*	4.37
Arid	*Wildlife Only*	*Phacelia* sp.	3.18
Arid	*Wildlife Only*	*Gilia tricolor*	2.68
Arid	*Wildlife Only*	*Acmispon wrangelianus*	2.43
Arid	*Wildlife Only*	*Festuca myuros*	2.37
Arid	*Wildlife Only*	*Medicago polymorpha*	2.11
Arid	*Wildlife Only*	*Bromus rubens*	1.77
Arid	*Wildlife Only*	*Medicago polymorpha*	1.64
Arid	*Wildlife Only*	*Avena fatua*	1.46
Arid	*Wildlife Only*	*Claytonia perfoliata*	1.44
Arid	*Wildlife Only*	Cerastium glomeratum	1.18
Arid	*No Herbivores*	*Bromus hordeaceus*	39.87
Arid	*No Herbivores*	*Bromus diandrus*	33.73
Arid	*No Herbivores*	*Hordeum murinum*	5.57
Arid	*No Herbivores*	*Trifolium* sp.	5.39
Arid	*No Herbivores*	*Festuca myuros*	2.97
Arid	*No Herbivores*	*Erodium* sp.	2.42
Arid	*No Herbivores*	*Bromus tectorum*	1.85
Arid	*All Herbivores*	*Bromus hordeaceus*	25.15
Arid	*All Herbivores*	*Erodium* sp.	15.59
Arid	*All Herbivores*	*Bromus tectorum*	9.39
Arid	*All Herbivores*	*Medicago polymorpha*	7.98

Arid	*All Herbivores*	*Bromus diandrus*	7.29
Arid	*All Herbivores*	*Acmispon wrangelianus*	5.36
Arid	*All Herbivores*	*Plagiobothrys nothofulvus*	5.30
Arid	*All Herbivores*	*Trifolium* sp.	4.22
Arid	*All Herbivores*	*Phacelia* sp.	2.84
Arid	*All Herbivores*	*Hordeum murinum*	2.60
Arid	*All Herbivores*	*Calandrinia menziesii*	1.87
Arid	*All Herbivores*	*Stellaria* sp.	1.87
Arid	*All Herbivores*	*Leptosiphon* sp.	1.79
Arid	*Wildlife Only*	*Bromus diandrus*	32.99
Arid	*Wildlife Only*	*Bromus hordeaceus*	20.39
Arid	*Wildlife Only*	*Trifolium* sp.	11.00
Arid	*Wildlife Only*	*Triteleia ixioides*	6.02
Arid	*Wildlife Only*	*Bromus tectorum*	4.49
Arid	*Wildlife Only*	*Acmispon wrangelianus*	3.10
Arid	*Wildlife Only*	*Hordeum murinum*	2.84
Arid	*Wildlife Only*	*Erodium* sp.	2.37
Arid	*Wildlife Only*	*Gilia tricolor*	2.34
Arid	*Wildlife Only*	*Phacelia* sp.	1.57
Arid	*Wildlife Only*	*Medicago polymorpha*	1.35
Arid	*Wildlife Only*	*Dichelostemma capitatum*	1.29
Arid	*Wildlife Only*	*Claytonia perfoliata*	1.18
Arid	*No Herbivores*	*Bromus diandrus*	42.30
Arid	*No Herbivores*	*Bromus hordeaceus*	34.91
Arid	*No Herbivores*	*Trifolium* sp.	4.49
Arid	*No Herbivores*	*Triteleia ixioides*	3.81
Arid	*No Herbivores*	*Hordeum murinum*	3.53
Arid	*No Herbivores*	*Festuca myuros*	1.89
Arid	*All Herbivores*	*Bromus hordeaceus*	29.79
Arid	*All Herbivores*	*Erodium* sp.	27.90
Arid	*All Herbivores*	*Medicago polymorpha*	8.84
Arid	*All Herbivores*	*Bromus diandrus*	6.91
Arid	*All Herbivores*	*Trifolium* sp.	5.21
Arid	*All Herbivores*	*Acmispon wrangelianus*	3.61
Arid	*All Herbivores*	*Triteleia ixioides*	3.08
Arid	*All Herbivores*	*Hordeum murinum*	2.10
Arid	*All Herbivores*	*Stellaria* sp.	1.71
Arid	*All Herbivores*	*Gilia tricolor*	1.53
Arid	*Wildlife Only*	*Bromus diandrus*	30.64
Arid	*Wildlife Only*	*Bromus hordeaceus*	21.31
Arid	*Wildlife Only*	*Trifolium* sp.	11.07
Arid	*Wildlife Only*	*Triteleia ixioides*	4.93
Arid	*Wildlife Only*	*Bromus tectorum*	4.85
Arid	*Wildlife Only*	*Hordeum murinum*	4.78

Arid	*Wildlife Only*	*Erodium* sp.	2.89
Arid	*Wildlife Only*	*Avena fatua*	2.79
Arid	*Wildlife Only*	*Gilia tricolor*	1.70
Arid	*Wildlife Only*	*Stipa pulchra*	1.58
Arid	*Wildlife Only*	*Medicago polymorpha*	1.32
Arid	*Wildlife Only*	*Acmispon wrangelianus*	1.27
Arid	*Wildlife Only*	*Medicago polymorpha*	1.08
Arid	*No Herbivores*	*Bromus hordeaceus*	43.41
Arid	*No Herbivores*	*Bromus diandrus*	26.53
Arid	*No Herbivores*	*Hordeum murinum*	6.54
Arid	*No Herbivores*	*Trifolium* sp.	5.55
Arid	*No Herbivores*	*Festuca myuros*	2.94
Arid	*No Herbivores*	*Erodium* sp.	2.31
Arid	*No Herbivores*	*Bromus tectorum*	1.84
Arid	*No Herbivores*	*Triteleia ixioides*	1.46
Intermediate	*All Herbivores*	*Bromus diandrus*	34.84
Intermediate	*All Herbivores*	*Ericameria nauseosa*	14.64
Intermediate	*All Herbivores*	*Bromus hordeaceus*	11.59
Intermediate	*All Herbivores*	*Erodium* sp.	9.23
Intermediate	*All Herbivores*	*Bromus tectorum*	6.12
Intermediate	*All Herbivores*	*Plagiobothrys nothofulvus*	4.61
Intermediate	*All Herbivores*	*Acmispon wrangelianus*	4.14
Intermediate	*All Herbivores*	*Bromus rubens*	3.90
Intermediate	*All Herbivores*	*Medicago polymorpha*	2.07
Intermediate	*Wildlife Only*	*Bromus diandrus*	41.73
Intermediate	*Wildlife Only*	*Bromus hordeaceus*	9.99
Intermediate	*Wildlife Only*	*Ericameria nauseosa*	9.97
Intermediate	*Wildlife Only*	*Ribes californicum* var. *hesperium*	5.68
Intermediate	*Wildlife Only*	*Nemophila menziesii*	5.55

Intermediate	*Wildlife Only*	*Bromus tectorum*	4.95
Intermediate	*Wildlife Only*	*Erodium* sp.	2.98
Intermediate	*Wildlife Only*	*Bromus rubens*	2.83
Intermediate	*Wildlife Only*	Asteraceae sp.	2.27
Intermediate	*Wildlife Only*	*Phacelia* sp.	1.95
Intermediate	*Wildlife Only*	*Gayophytum diffusum* ssp. *parviflorum*	1.84
Intermediate	*Wildlife Only*	*Festuca myuros*	1.63
Intermediate	*No Herbivores*	*Bromus diandrus*	41.05
Intermediate	*No Herbivores*	*Ericameria nauseosa*	16.98
Intermediate	*No Herbivores*	*Bromus rubens*	9.81
Intermediate	*No Herbivores*	*Bromus tectorum*	8.38
Intermediate	*No Herbivores*	*Erodium* sp.	6.92
Intermediate	*No Herbivores*	*Bromus hordeaceus*	5.38
Intermediate	*No Herbivores*	*Gayophytum diffusum* ssp. *parviflorum*	2.20
Intermediate	*All Herbivores*	*Ericameria nauseosa*	27.07
Intermediate	*All Herbivores*	*Bromus hordeaceus*	16.72
Intermediate	*All Herbivores*	*Bromus tectorum*	13.21
Intermediate	*All Herbivores*	*Bromus diandrus*	12.06
Intermediate	*All Herbivores*	*Bromus rubens*	5.63
Intermediate	*All Herbivores*	*Erodium* sp.	4.60
Intermediate	*All Herbivores*	*Festuca myuros*	3.27
Intermediate	*All Herbivores*	*Gayophytum diffusum* ssp. *parviflorum*	2.68

Intermediate	*All Herbivores*	*Acmispon wrangelianus*	2.60
Intermediate	*All Herbivores*	*Urtica dioica*	1.74
Intermediate	*All Herbivores*	*Medicago polymorpha*	1.42
Intermediate	*Wildlife Only*	*Bromus diandrus*	61.14
Intermediate	*Wildlife Only*	*Bromus hordeaceus*	9.92
Intermediate	*Wildlife Only*	*Bromus tectorum*	5.88
Intermediate	*Wildlife Only*	*Erodium* sp.	5.18
Intermediate	*Wildlife Only*	*Urtica dioica*	3.19
Intermediate	*Wildlife Only*	*Ericameria nauseosa*	2.78
Intermediate	*Wildlife Only*	*Bromus rubens*	2.17
Intermediate	*No Herbivores*	*Bromus diandrus*	42.98
Intermediate	*No Herbivores*	*Ericameria nauseosa*	15.32
Intermediate	*No Herbivores*	*Erodium* sp.	6.68
Intermediate	*No Herbivores*	*Bromus hordeaceus*	6.63
Intermediate	*No Herbivores*	*Festuca myuros*	5.77
Intermediate	*No Herbivores*	*Bromus tectorum*	4.37
Intermediate	*No Herbivores*	*Bromus rubens*	3.44
Intermediate	*No Herbivores*	*Acmispon wrangelianus*	2.04
Intermediate	*No Herbivores*	*Viola purpurea*	2.04
Intermediate	*No Herbivores*	*Gayophytum diffusum* ssp. *parviflorum*	1.78
Intermediate	*All Herbivores*	*Ericameria nauseosa*	38.24
Intermediate	*All Herbivores*	*Bromus diandrus*	12.48

Intermediate	*All Herbivores*	*Bromus tectorum*	11.92
Intermediate	*All Herbivores*	*Bromus hordeaceus*	10.74
Intermediate	*All Herbivores*	*Erodium* sp.	9.34
Intermediate	*All Herbivores*	*Bromus rubens*	7.16
Intermediate	*All Herbivores*	*Hordeum murinum*	1.48
Intermediate	*Wildlife Only*	*Bromus diandrus*	40.54
Intermediate	*Wildlife Only*	*Ericameria nauseosa*	20.30
Intermediate	*Wildlife Only*	*Bromus tectorum*	10.71
Intermediate	*Wildlife Only*	*Bromus hordeaceus*	6.47
Intermediate	*Wildlife Only*	*Erodium* sp.	5.40
Intermediate	*Wildlife Only*	*Festuca myuros*	3.37
Intermediate	*Wildlife Only*	*Bromus rubens*	3.19
Intermediate	*Wildlife Only*	*Gayophytum diffusum* ssp. *parviflorum*	3.19
Intermediate	*No Herbivores*	*Bromus diandrus*	46.13
Intermediate	*No Herbivores*	*Ericameria nauseosa*	26.39
Intermediate	*No Herbivores*	*Bromus hordeaceus*	8.49
Intermediate	*No Herbivores*	*Bromus tectorum*	5.89
Intermediate	*No Herbivores*	*Erodium* sp.	4.34
Mesic	*All Herbivores*	*Ribes californicum* var. *hesperium*	30.67
Mesic	*All Herbivores*	*Ranunculus californicus*	13.24
Mesic	*All Herbivores*	*Bromus tectorum*	10.25
Mesic	*All Herbivores*	*Collinsia parviflora*	10.25
Mesic	*All Herbivores*	*Melica californica*	6.36
Mesic	*All Herbivores*	*Gayophytum diffusum* ssp. *parviflorum*	5.73
Mesic	*All Herbivores*	*Acmispon wrangelianus*	5.00
Mesic	*All Herbivores*	*Galium aparine*	4.83

Mesic	*All Herbivores*	Symphoricarpos mollis	3.30
Mesic	*All Herbivores*	*Mimulus subsecundus*	2.85
Mesic	*Wildlife Only*	*Ribes californicum* var. *hesperium*	29.07
Mesic	*Wildlife Only*	*Galium aparine*	17.05
Mesic	*Wildlife Only*	*Bromus tectorum*	8.25
Mesic	*Wildlife Only*	*Melica californica*	6.00
Mesic	*Wildlife Only*	Symphoricarpos mollis	5.78
Mesic	*Wildlife Only*	*Hosackia crassifolia* var. *crassifolia*	4.12
Mesic	*Wildlife Only*	*Phacelia* sp.	3.75
Mesic	*Wildlife Only*	*Gayophytum diffusum* ssp. *parviflorum*	3.14
Mesic	*Wildlife Only*	*Mimulus subsecundus*	2.72
Mesic	*Wildlife Only*	*Plagiobothrys nothofulvus*	2.69
Mesic	*Wildlife Only*	*Collinsia childii*	2.65
Mesic	*Wildlife Only*	*Pholistoma auritum*	2.49
Mesic	*Wildlife Only*	*Acmispon wrangelianus*	2.35
Mesic	*No Herbivores*	*Ribes californicum* var. *hesperium*	35.94
Mesic	*No Herbivores*	*Galium aparine*	14.19
Mesic	*No Herbivores*	Symphoricarpos mollis	8.36
Mesic	*No Herbivores*	*Collinsia childii*	7.36
Mesic	*No Herbivores*	*Melica californica*	5.33
Mesic	*No Herbivores*	*Bromus tectorum*	5.31
Mesic	*No Herbivores*	*Hosackia crassifolia* var. *crassifolia*	4.48
Mesic	*No Herbivores*	*Claytonia perfoliata*	3.12
Mesic	*No Herbivores*	*Mimulus subsecundus*	2.30
Mesic	*No Herbivores*	*Acmispon wrangelianus*	2.09
Mesic	*No Herbivores*	*Plagiobothrys nothofulvus*	2.01
Mesic	*All Herbivores*	*Ribes californicum* var. *hesperium*	37.87
Mesic	*All Herbivores*	*Ranunculus californicus*	17.10
Mesic	*All Herbivores*	*Bromus tectorum*	8.65
Mesic	*All Herbivores*	*Collinsia parviflora*	7.46
Mesic	*All Herbivores*	*Melica californica*	6.85
Mesic	*All Herbivores*	*Gayophytum diffusum* ssp. *parviflorum*	5.43
Mesic	*All Herbivores*	*Acmispon wrangelianus*	3.33
Mesic	*All Herbivores*	*Plagiobothrys nothofulvus*	2.87
Mesic	*All Herbivores*	*Mimulus subsecundus*	2.41
Mesic	*Wildlife Only*	*Ribes californicum* var. *hesperium*	30.82
Mesic	*Wildlife Only*	*Galium aparine*	17.60
Mesic	*Wildlife Only*	Symphoricarpos mollis	15.54
Mesic	*Wildlife Only*	*Bromus tectorum*	3.99
Mesic	*Wildlife Only*	*Melica californica*	3.20
Mesic	*Wildlife Only*	*Pholistoma auritum*	3.20
Mesic	*Wildlife Only*	*Phacelia* sp.	2.98

Mesic	*Wildlife Only*	*Plagiobothrys nothofulvus*	2.94
Mesic	*Wildlife Only*	*Collinsia childii*	2.75
Mesic	*Wildlife Only*	*Gayophytum diffusum* ssp. *parviflorum*	2.08
Mesic	*Wildlife Only*	*Claytonia perfoliata*	2.02
Mesic	*Wildlife Only*	*Acmispon wrangelianus*	1.59
Mesic	*Wildlife Only*	*Hosackia crassifolia* var. *crassifolia*	1.52
Mesic	*No Herbivores*	*Ribes californicum* var. *hesperium*	29.92
Mesic	*No Herbivores*	*Galium aparine*	18.78
Mesic	*No Herbivores*	Symphoricarpos mollis	10.85
Mesic	*No Herbivores*	*Collinsia childii*	9.62
Mesic	*No Herbivores*	*Bromus tectorum*	5.47
Mesic	*No Herbivores*	*Melica californica*	4.38
Mesic	*No Herbivores*	*Plagiobothrys nothofulvus*	2.55
Mesic	*No Herbivores*	*Acmispon wrangelianus*	2.40
Mesic	*No Herbivores*	*Penstemon laetus*	1.75
Mesic	*No Herbivores*	*Claytonia perfoliata*	1.69
Mesic	*No Herbivores*	*Dichelostemma capitatum*	1.56
Mesic	*No Herbivores*	*Clarkia unguiculata*	1.15
Mesic	*All Herbivores*	*Ribes californicum* var. *hesperium*	29.05
Mesic	*All Herbivores*	*Ranunculus californicus*	12.85
Mesic	*All Herbivores*	*Acmispon wrangelianus*	9.03
Mesic	*All Herbivores*	*Gayophytum diffusum* ssp. *parviflorum*	6.53
Mesic	*All Herbivores*	*Collinsia parviflora*	6.16
Mesic	*All Herbivores*	*Bromus tectorum*	5.61
Mesic	*All Herbivores*	Symphoricarpos mollis	5.49
Mesic	*All Herbivores*	*Galium aparine*	5.13
Mesic	*All Herbivores*	*Prunus virginiana*	3.66
Mesic	*All Herbivores*	*Mimulus subsecundus*	2.04
Mesic	*All Herbivores*	*Festuca myuros*	2.01
Mesic	*All Herbivores*	*Plagiobothrys nothofulvus*	1.95
Mesic	*All Herbivores*	*Melica californica*	1.62
Mesic	*Wildlife Only*	*Ribes californicum* var. *hesperium*	24.15
Mesic	*Wildlife Only*	*Galium aparine*	18.74
Mesic	*Wildlife Only*	*Penstemon* sp.	5.74
Mesic	*Wildlife Only*	*Prunus virginiana*	5.27
Mesic	*Wildlife Only*	*Acmispon wrangelianus*	4.81
Mesic	*Wildlife Only*	*Mimulus subsecundus*	4.74
Mesic	*Wildlife Only*	Symphoricarpos mollis	4.39
Mesic	*Wildlife Only*	*Gayophytum diffusum* ssp. *parviflorum*	3.54
Mesic	*Wildlife Only*	*Hosackia crassifolia* var. *crassifolia*	2.71
Mesic	*Wildlife Only*	*Melica californica*	2.58

Mesic	*Wildlife Only*	*Phacelia* sp.	2.18
Mesic	*Wildlife Only*	*Stipa pulchra*	2.16
Mesic	*Wildlife Only*	*Pholistoma auritum*	2.11
Mesic	*Wildlife Only*	*Claytonia perfoliata*	1.94
Mesic	*Wildlife Only*	*Festuca myuros*	1.91
Mesic	*Wildlife Only*	*Leptosiphon* sp.	1.78
Mesic	*Wildlife Only*	*Bromus tectorum*	1.63
Mesic	*No Herbivores*	*Ribes californicum* var. *hesperium*	23.96
Mesic	*No Herbivores*	*Galium aparine*	20.15
Mesic	*No Herbivores*	*Penstemon* sp.	8.73
Mesic	*No Herbivores*	*Bromus tectorum*	4.60
Mesic	*No Herbivores*	*Plagiobothrys nothofulvus*	4.60
Mesic	*No Herbivores*	*Prunus virginiana*	3.65
Mesic	*No Herbivores*	*Melica californica*	3.63
Mesic	*No Herbivores*	*Mimulus subsecundus*	3.47
Mesic	*No Herbivores*	*Acmispon wrangelianus*	3.09
Mesic	*No Herbivores*	Symphoricarpos mollis	3.03
Mesic	*No Herbivores*	*Clarkia unguiculata*	2.83
Mesic	*No Herbivores*	*Stipa pulchra*	2.69
Mesic	*No Herbivores*	*Pholistoma auritum*	2.62
Mesic	*No Herbivores*	*Claytonia perfoliata*	1.93
Mesic	*No Herbivores*	*Gayophytum diffusum* ssp. *parviflorum*	1.67

Table S5. Additional data sources.

Climate	Treatment	Species Name	LA, SLA, and LDMC Data Sources
Arid	*All Herbivores*	*Bromus tectorum*	2019 trait collection
Arid	*Wildlife Only*	*Claytonia perfoliata*	LA and SLA from Kattge et al., 2020
Arid	*Wildlife Only*	*Claytonia perfoliata*	LA and SLA from Kattge et al., 2020
Arid	*Wildlife Only*	*Dichelostemma capitatum*	LA, SLA, and LDMC from Kattge et al., 2020
Arid	*Wildlife Only*	*Gilia tricolor*	SLA from arid—*All Herbivores* treatment plots
Arid	*Wildlife Only*	*Gilia tricolor*	SLA from arid—*All Herbivores* treatment plots
Arid	*Wildlife Only*	*Gilia tricolor*	SLA from arid—*All Herbivores* treatment plots
Arid	*Wildlife Only*	*Hordeum murinum*	LA from 2019 trait collection
Arid	*Wildlife Only*	*Hordeum murinum*	LA from 2019 trait collection
Arid	*Wildlife Only*	*Hordeum murinum*	LA from 2019 trait collection
Arid	*All Herbivores*	*Leptosiphon* sp.	LA estimate from Lambrecht 2013 field measurements of L. bicolor; SLA from TRY DB records for *L. bicolor*
Arid	*All Herbivores*	*Leptosiphon* sp.	LA estimate from Lambrecht 2013 field measurements of *Leptosiphon bicolor*; SLA from Kattge et al., 2020, records for *L. bicolor*
Arid	*Wildlife Only*	*Medicago polymorpha*	LDMC from Arid—*All Herbivores*
Arid	*Wildlife Only*	*Medicago polymorpha*	LDMC from Arid—*All Herbivores*
Arid	*Wildlife Only*	*Medicago polymorpha*	LDMC from Arid—*All Herbivores*
Arid	*Wildlife Only*	*Phacelia* sp.	Trait data from Kattge et al., 2020 for *Phacelia tanacetifolia*
Arid	*All Herbivores*	*Phacelia* sp.	Trait data from Kattge et al., 2020 for *Phacelia tanacetifolia*
Arid	*Wildlife Only*	*Phacelia* sp.	Trait data from Kattge et al., 2020 for *Phacelia tanacetifolia*
Arid	*All Herbivores*	*Stellaria* sp.	Trait data from Kattge et al., 2020 for *Stellaria media*

Arid	*All Herbivores*	*Stellaria* sp.	Trait data from Kattge et al., 2020 for *Stellaria media*
Arid	*Wildlife Only*	*Stipa pulchra*	Trait data from Kattge et al., 2020 for *Stellaria media*
Intermediate	*All Herbivores*	*Acmispon wrangelianus*	SLA and LDMC from Kattge et al., 2020; LA from average of arid and mesic climate level collections
Intermediate	*All Herbivores*	*Acmispon wrangelianus*	SLA and LDMC from Kattge et al., 2020; LA from average of arid and mesic climate level collections
Intermediate	*No Herbivores*	*Acmispon wrangelianus*	SLA and LDMC from Kattge et al., 2020; LA from average of arid and mesic climate level collections
Intermediate	*Wildlife Only*	Asteraceae sp.	Trait data from Kattge et al. 2020
Intermediate	*Wildlife Only*	*Bromus diandrus*	LA and SLA take from intermediate–*Wildlife Only*
Intermediate	*Wildlife Only*	*Bromus diandrus*	LA and SLA take from intermediate–*Wildlife Only*
Intermediate	*Wildlife Only*	*Bromus diandrus*	LA and SLA take from intermediate–*Wildlife Only*
Intermediate	*All Herbivores*	*Bromus rubens*	LA and LDMC from arid climate level; SLA from Kattge et al. 2020
Intermediate	*Wildlife Only*	*Bromus rubens*	LA and LDMC from arid climate level; SLA from Kattge et al. 2020
Intermediate	*No Herbivores*	*Bromus rubens*	LA and LDMC from arid climate level; SLA from Kattge et al. 2020
Intermediate	*All Herbivores*	*Bromus rubens*	LA and LDMC from arid climate level; SLA from Kattge et al. 2020
Intermediate	*Wildlife Only*	*Bromus rubens*	LA and LDMC from arid climate level; SLA from Kattge et al. 2020
Intermediate	*No Herbivores*	*Bromus rubens*	LA and LDMC from arid climate level; SLA from Kattge et al. 2020
Intermediate	*All Herbivores*	*Bromus rubens*	LA and LDMC from arid climate level; SLA from Kattge et al. 2020
Intermediate	*Wildlife Only*	*Bromus rubens*	LA and LDMC from arid climate level; SLA from Kattge et al. 2020
Intermediate	*All Herbivores*	*Bromus tectorum*	2019 trait collection
Intermediate	*Wildlife Only*	*Bromus tectorum*	2019 trait collection
Intermediate	*No Herbivores*	*Bromus tectorum*	2019 trait collection
Intermediate	*All Herbivores*	*Bromus tectorum*	2019 trait collection

Intermediate	*Wildlife Only*	*Bromus tectorum*	2019 trait collection
Intermediate	*No Herbivores*	*Bromus tectorum*	2019 trait collection
Intermediate	*All Herbivores*	*Bromus tectorum*	2019 trait collection
Intermediate	*Wildlife Only*	*Bromus tectorum*	2019 trait collection
Intermediate	*No Herbivores*	*Bromus tectorum*	2019 trait collection
Intermediate	*Wildlife Only*	*Phacelia* sp.	Trait data from Kattge et al. 2020 for *Phacelia tanacetifolia*
Intermediate	*All Herbivores*	*Plagiobothrys nothofulvus*	Intermediate–*All Herbivores*
Intermediate	*Wildlife Only*	*Ribes californicum* var. *hesperium*	Intermediate–*All Herbivores*
Intermediate	*All Herbivores*	*Urtica dioica*	LA and SLA from Kattge et al. 2020
Intermediate	*Wildlife Only*	*Urtica dioica*	LA and SLA from Kattge et al. 2020
Intermediate	*No Herbivores*	*Viola purpurea*	Trait data from Kattge et al. 2020 for *Viola adunca*
Mesic	*All Herbivores*	*Bromus tectorum*	2019 trait collection
Mesic	*Wildlife Only*	*Bromus tectorum*	Trait data from Kattge et al. 2020
Mesic	*No Herbivores*	*Bromus tectorum*	2019 trait collection
Mesic	*All Herbivores*	*Bromus tectorum*	2019 trait collection
Mesic	*Wildlife Only*	*Bromus tectorum*	Trait data from Kattge et al. 2020
Mesic	*No Herbivores*	*Bromus tectorum*	2019 trait collection
Mesic	*All Herbivores*	*Bromus tectorum*	2019 trait collection
Mesic	*Wildlife Only*	*Bromus tectorum*	Trait data from Kattge et al. 2020
Mesic	*No Herbivores*	*Bromus tectorum*	2019 trait collection
Mesic	*No Herbivores*	*Clarkia unguiculata*	SLA and LDMC from Kattge et al. 2020 for *Clarkia purpurea*; LA estimate from Jona and Geber 1999

Mesic	*No Herbivores*	*Clarkia unguiculata*	SLA and LDMC from Kattge et al. 2020 for *Clarkia purpurea*; LA estimate from Jona and Geber 1999
Mesic	*No Herbivores*	*Claytonia perfoliata*	LA and SLA from Kattge et al. 2020
Mesic	*Wildlife Only*	*Claytonia perfoliata*	LA and SLA from Kattge et al. 2020
Mesic	*No Herbivores*	*Claytonia perfoliata*	LA and SLA from Kattge et al. 2020
Mesic	*Wildlife Only*	*Claytonia perfoliata*	LA and SLA from Kattge et al. 2020
Mesic	*No Herbivores*	*Claytonia perfoliata*	LA and SLA from Kattge et al. 2020
Mesic	*Wildlife Only*	*Collinsia childii*	Collection at Intermediate–*Wildlife Only*
Mesic	*No Herbivores*	*Collinsia childii*	Collection at Intermediate–*Wildlife Only*
Mesic	*Wildlife Only*	*Collinsia childii*	Collection at Intermediate–*Wildlife Only*
Mesic	*No Herbivores*	*Collinsia childii*	Collection at Intermediate–*Wildlife Only*
Mesic	*All Herbivores*	*Collinsia parviflora*	Collection at Intermediate–*Wildlife Only*
Mesic	*All Herbivores*	*Collinsia parviflora*	Collection at Intermediate–*Wildlife Only*
Mesic	*All Herbivores*	*Collinsia parviflora*	Collection at Intermediate–*Wildlife Only*
Mesic	*No Herbivores*	*Dichelostemma capitatum*	Trait data from Kattge et al. 2020
Mesic	*All Herbivores*	*Festuca myuros*	2019 trait collection
Mesic	*Wildlife Only*	*Festuca myuros*	2019 trait collection
Mesic	*No Herbivores*	*Hosackia crassifolia* var. *crassifolia*	Collection at Mesic–*Wildlife Only*
Mesic	*Wildlife Only*	*Leptosiphon* sp.	LA estimate from Lambrecht 2013 field measurements of L*eptosiphon bicolor*; SLA from Kattge et al., 2020 of *L. bicolor*
Mesic	*All Herbivores*	*Mimulus subsecundus*	SLA from Kattge et al. 2020 for *Mimulus douglasii*
Mesic	*Wildlife Only*	*Mimulus subsecundus*	SLA from Kattge et al. 2020 for *Mimulus douglasii*
Mesic	*No Herbivores*	*Mimulus subsecundus*	SLA from Kattge et al. 2020 for *Mimulus douglasii*

Mesic	*All Herbivores*	*Mimulus subsecundus*	SLA from Kattge et al. 2020 for *Mimulus douglasii*
Mesic	*All Herbivores*	*Mimulus subsecundus*	SLA from Kattge et al. 2020 for *Mimulus douglasii*
Mesic	*Wildlife Only*	*Mimulus subsecundus*	SLA from Kattge et al. 2020 for *Mimulus douglasii*
Mesic	*No Herbivores*	*Mimulus subsecundus*	SLA from Kattge et al. 2020 for *Mimulus douglasii*
Mesic	*No Herbivores*	*Penstemon laetus*	LA and SLA values from Kattge et al., 2020, from records for all North American *Penstemon* species
Mesic	*Wildlife Only*	*Penstemon* sp.	LA and SLA values from Kattge et al., 2020, from records for all North American *Penstemon* species
Mesic	*No Herbivores*	*Penstemon* sp.	LA and SLA values from Kattge et al., 2020, from records for all North American *Penstemon* species
Mesic	*Wildlife Only*	*Phacelia* sp.	Trait data from Kattge et al. 2020 for *Phacelia tanacetifolia*
Mesic	*Wildlife Only*	*Phacelia* sp.	Trait data from Kattge et al. 2020 for *Phacelia tanacetifolia*
Mesic	*Wildlife Only*	*Phacelia* sp.	Trait data from Kattge et al. 2020 for *Phacelia tanacetifolia*
Mesic	*All Herbivores*	*Prunus virginiana*	LA and SLA from Kattge et al., 2020, for *Prunus virginian*a; LDMC from Kattge et al., 2020, for *Prunus fasciculata*
Mesic	*Wildlife Only*	*Prunus virginiana*	LA and SLA from Kattge et al., 2020, for *Prunus virginian*a; LDMC from Kattge et al., 2020, for *Prunus fasciculata*
Mesic	*No Herbivores*	*Prunus virginiana*	LA and SLA from Kattge et al., 2020, for *Prunus virginian*a; LDMC from Kattge et al., 2020, for *Prunus fasciculata*
Mesic	*Wildlife Only*	*Stipa pulchra*	Trait data from Kattge et al., 2020 for *Stipa* genus records
Mesic	*No Herbivores*	*Stipa pulchra*	Trait data from Kattge et al., 2020 for *Stipa* genus records

Species were not collected if they were too rare or not evenly dispersed enough throughout the plot to warrant collection. Seed mass and leaf nitrogen content (LNC) values were obtained from Try Plant Trait Database (Kattge et al. 2020) for the same species unless otherwise noted, and all records were averaged from available public sources. Table notes where substitutions were made when specific plant species had no data records. When choosing species for substitution, we prioritized using data collected in 2019 or from another replicate of the same climate and herbivore treatment. If unavailable, we searched for data for species of the same genus that grew in the region or chose members of the same genus outside the area or from the family level.

Models, AIC Tables, and Summaries

Table S6. *Community-weighted means (CWMs) linear mixed effect models, Akaike information criterion (AIC) and degrees of freedom (df). The fixed effects are herbivory treatment and climate level with a random effect of "plot", which was one of the twenty-seven treatment locations from where samples were collected (plot location = PL). Final best-fit models are in bold.*

Leaf Area$_{CWM}$

<table>
<tr><th>Model</th><th>df</th><th>AICc</th></tr>
<tr><td>LA$_{CWM}$ ~ climate * treatment + (1|PL)</td><td>11</td><td>25.58</td></tr>
<tr><td>LA$_{CWM}$ ~ climate + treatment + (1|PL)</td><td>7</td><td>53.28</td></tr>
<tr><td>LA$_{CWM}$ ~ climate + (1|PL)</td><td>5</td><td>48.77</td></tr>
<tr><td>LA$_{CWM}$ ~ treatment + (1|PL)</td><td>5</td><td>52.22</td></tr>
<tr><td>LA$_{CWM}$ ~ 1 + (1|PL)</td><td>3</td><td>48.45</td></tr>
</table>

Specific Leaf Area$_{CWM}$

<table>
<tr><th>Model</th><th>df</th><th>AICc</th></tr>
<tr><td>SLA ~ climate * treatment + (1|PL)</td><td>11</td><td>60.65</td></tr>
<tr><td>SLA$_{CWM}$ ~ climate + treatment + (1|PL)</td><td>7</td><td>68.07</td></tr>
<tr><td>SLA$_{CWM}$ ~ climate + (1|PL)</td><td>5</td><td>71.66</td></tr>
<tr><td>SLA$_{CWM}$ ~ treatment + (1|PL)</td><td>5</td><td>76.30</td></tr>
<tr><td>SLA$_{CWM}$ ~ 1 + (1|PL)</td><td>3</td><td>78.10</td></tr>
</table>

Leaf Dry Matter Content$_{CWM}$

<table>
<tr><th>Model</th><th>df</th><th>AICc</th></tr>
<tr><td>LDMC$_{CWM}$ ~ climate * treatment + (1|PL)</td><td>11</td><td>215.19</td></tr>
<tr><td>LDMC$_{CWM}$ ~ climate + treatment + (1|PL)</td><td>7</td><td>208.87</td></tr>
<tr><td>LDMC$_{CWM}$ ~ climate + (1|PL)</td><td>5</td><td>214.22</td></tr>
<tr><td>LDMC$_{CWM}$ ~ treatment + (1|PL)</td><td>5</td><td>209.84</td></tr>
<tr><td>LDMC$_{CWM}$ ~ 1 + (1|PL)</td><td>3</td><td>215.61</td></tr>
</table>

Seed Mass$_{CWM}$

<table>
<tr><th>Model</th><th>df</th><th>AICc</th></tr>
<tr><td>Seed mass$_{CWM}$ ~ climate * treatment + (1|PL)</td><td>11</td><td>40.37</td></tr>
<tr><td>Seed mass$_{CWM}$ ~ climate + treatment + (1|PL)</td><td>7</td><td>31.31</td></tr>
<tr><td>Seed mass$_{CWM}$ ~ climate + (1|PL)</td><td>5</td><td>43.80</td></tr>
<tr><td>Seed mass$_{CWM}$ ~ treatment + (1|PL)</td><td>5</td><td>38.35</td></tr>
<tr><td>Seed mass$_{CWM}$ ~ 1 + (1|PL)</td><td>3</td><td>47.91</td></tr>
</table>

Leaf Nitrogen Content$_{CWM}$

<table>
<tr><th>Model</th><th>df</th><th>AICc</th></tr>
<tr><td>LNC$_{CWM}$ ~ climate * treatment + (1|PL)</td><td>11</td><td>63.64</td></tr>
<tr><td>LNC$_{CWM}$ ~ climate + treatment + (1|PL)</td><td>7</td><td>52.52</td></tr>
<tr><td>LNC$_{CWM}$ ~ climate + (1|PL)</td><td>5</td><td>60.76</td></tr>
<tr><td>LNC$_{CWM}$ ~ treatment + (1|PL)</td><td>5</td><td>57.12</td></tr>
<tr><td>LNC$_{CWM}$ ~ 1 + (1|PL)</td><td>3</td><td>61.72</td></tr>
</table>

Table S7. *Community-weighted means (CWMs) linear mixed effect models conditional and marginal R2.*

Final Model	Marginal R2	Conditional R2
Leaf Area (LA_{CWM})	0.876	0.876
Specific Leaf Area (SLA_{CWM})	0.850	0.850
Leaf Dry Mass Content ($LDMC_{CWM}$)	0.285	0.544
Seed $Mass_{CWM}$	0.674	0.674
Leaf Nitrogen Content (LNC_{CWM})	0.506	0.506

Table S8. *Functional diversity metrics linear mixed effect models, Akaike information criterion (AIC) and degrees of freedom (df). The fixed effects are herbivory treatment and climate level with a random effect of "plot", which was one of the twenty-seven treatment locations from where samples were collected (plot location = PL). Final best-fit models are in bold.*

**Functional Richness (FRic)**			**Functional Evenness (FEve)**		
Model	*df*	*AICc*	*Model*	*df*	*AICc*
FRic ~ climate * treatment + (1|PL)	11	150.60	FEve ~ climate * treatment + (1|PL)	11	-14.30
FRic ~ climate + treatment + (1|PL)	7	136.68	FEve ~ climate + treatment + (1|PL)	7	-28.17
FRic ~ climate + (1|PL)	5	130.59	FEve ~ climate + (1|PL)	5	-29.60
FRic ~ treatment + (1|PL)	5	133.42	FEve ~ treatment + (1|PL)	5	-34.67
**FRic ~ 1 + (1|PL)**	**3**	**128.56**	**FEve ~ 1 + (1|PL)**	**3**	**-34.98**
**Functional Diversity (FDiv)**			**Functional Dispersion (FDis)**		
Model	*df*	*AICc*	*Model*	*df*	*AICc*
**FDiv ~ climate * treatment + (1|PL)**	**11**	**-51.37**	**FDis ~ climate * treatment + (1|PL)**	**11**	**-8.57**
FDiv ~ climate + treatment + (1|PL)	7	-41.69	FDis ~ climate + treatment + (1|PL)	7	19.70
FDiv ~ climate + (1|PL)	5	-33.22	FDis ~ climate + (1|PL)	5	20.81
FDiv ~ treatment + (1|PL)	5	-45.20	FDis ~ treatment + (1|PL)	5	17.47
FDiv ~ 1 + (1|PL)	3	-37.00	FDis ~ 1 + (1|PL)	3	18.63

Table S9. *Functional diversity metrics linear mixed effect models conditional and marginal R2.*

**Final Model**	**Marginal R2**	**Conditional R2**
Functional Richness	0	0.335
Functional Evenness	0	5.291e-10
Functional Divergence	0.798	0.885
Functional Dispersion	0.894	0.894

Table S10. *ANOVA and PERMANOVA tables for functional trait community-weighted means (CWMs) specific averages, fixed averages, and intraspecifc trait variation (ITV).*

A. Specific Averages

Trait	Source of Variation	df	SS	MS	F	p
Leaf Area$_{CWM}$	climate	2	1769.9	884.97	10.737	<0.001
	treatment	2	971.6	485.8	5.894	0.01
	climate:treatment	4	4781.5	1195.38	14.503	<0.00001
	residuals	18	1483.7	82.43		
Specific Leaf Area$_{CWM}$	climate	2	8.2533	4.1267	21.5465	<0.00001
	treatment	2	4.5392	2.2696	11.8501	<0.0001
	climate:treatment	4	5.9698	1.4924	7.7925	<0.001
	Residuals	18	3.4474	0.1915		
Leaf Dry Matter Content$_{CWM}$	climate	2	889.71	444.85	6.9647	<0.01
	treatment	2	1006.03	503.02	7.8753	<0.01
	climate:treatment	4	584.8	146.2	2.2889	0.10
	residuals	22	1149.71	63.87		

B. Fixed Averages

Trait	Source of Variation	df	SS	MS	F	p
Leaf Area$_{CWM}$	climate	2	550.58	275.29	3.7	<0.05
	treatment	2	337	168.5	2.265	0.13
	climate:treatment	4	1650.65	412.66	5.546	<0.01
	residuals	18	1339.29	74.4		
Specific Leaf Area$_{CWM}$	climate	2	7.3471	3.6735	21.0642	<0.0001
	treatment	2	3.5235	1.7618	10.102	<0.01
	climate:treatment	4	3.6179	0.9045	5.1863	<0.01
	Residuals	18	3.1391	0.1744		
Leaf Dry Matter Content$_{CWM}$	climate	2	617.64	308.818	5.6673	0.01
	treatment	2	584.63	292.313	5.3644	0.01
	climate:treatment	4	589.25	147.314	2.7034	0.06
	residuals	22	980.84	54.491		

C. ITV Averages

Trait	Source of Variation	df	SS	MS	F	p
Leaf Area$_{CWM}$	climate	2	1647.46	823.73	55.508	2.0e-08
	treatment	2	176.28	88.14	5.939	0.01
	climate:treatment	4	1403.23	350.81	23.640	5.8e-07
	residuals	18	267.12	14.84		
Specific Leaf Area$_{CWM}$	climate	2	0.48236	0.241178	75.4	1.8e-09
	treatment	2	0.12616	0.06308	19.721	2.9e-05
	climate:treatment	4	0.66207	0.165517	51.746	1.2e-09
	residuals	18	0.05758	0.003199		
Leaf Dry Matter Content$_{CWM}$	climate	2	28.762	14.381	12.1007	<0.001
	treatment	2	70.427	35.214	29.6302	2.0e-06
	climate:treatment	4	12.832	3.208	2.6993	0.06
	residuals	22	21.392	1.188		

A. Specific averages, B. Fixed averages, and C. ITV. Tables include degrees of freedom (df), sum of squares (SS), mean sum of squares (MS), F-values, and p-values. Leaf area CWM was calculated with a PERMANOVA; all other traits were calculated with ANOVAs.
